# Greater physical fitness (Vo2Max) in healthy older adults associated with increased integrity of the Locus Coeruleus-Noradrenergic system

**DOI:** 10.21203/rs.3.rs-2556690/v2

**Published:** 2023-09-12

**Authors:** Emanuele RG Plini, Michael C Melnychuk, Ralph Andrews, Rory Boyle, Robert Whelan, Jeffrey S. Spence, Sandra B. Chapman, Ian H Robertson, Paul M Dockree

**Affiliations:** 1 Department of Psychology, Trinity College Institute of Neuroscience, Trinity College Dublin, Llyod Building, 42A Pearse St, 8PVX+GJ Dublin, Ireland; 2 Department of Neurology, Massachusetts General Hospital, Harvard Medical School, Building 149, Charlestown MA, USA; 3 Center for BrainHealth, The University of Texas at Dallas, Dallas, TX, USA,; 4 Department of Psychology, Global Brain Health Institute, Trinity College Dublin, Lloyd Building, 42A Pearse St, 8PVX+GJ Dublin, *Ireland*

**Keywords:** Locus Coeruleus, Vo2Max, aging, brain age, neuroimaging, voxel based morphometry

## Abstract

Physical activity (PA) is a key component for brain health and Reserve, and it is among the main dementia protective factors. However, the neurobiological mechanisms underpinning Reserve are not fully understood. In this regard, **a noradrenergic (NA) theory of cognitive reserve** (Robertson, 2013) **has** proposed that the upregulation of NA system might be a key factor for building reserve and resilience to neurodegeneration because of the neuroprotective role of NA across the brain. PA elicits an enhanced catecholamine response, in particular for NA. By increasing physical commitment, a greater amount of NA is synthetised in response to higher oxygen demand. More physically trained individuals show greater capabilities to carry oxygen resulting in greater Vo2max – a measure of oxygen uptake and physical fitness (PF). In the current study, we hypothesised that greater Vo2 max would be related to greater Locus Coeruleus (LC) MRI signal intensity. As hypothesised, greater Vo2max related to greater LC signal intensity across 41 healthy adults (age range 60–72). As a control procedure, in which these analyses were repeated for the other neuromodulators’ seeds (for Serotonin, Dopamine and Acetylcholine), weaker associations emerged. This newly established link between Vo2max and LC-NA system offers further understanding of the neurobiology underpinning Reserve in relationship to PA. While this study supports Robertson’s theory proposing the upregulation of the noradrenergic system as a possible key factor building Reserve, it also provide grounds for increasing LC-NA system resilience to neurodegeneration via Vo2max enhancement.

## Introduction

It is estimated that 40% of dementia causes are modifiable by simply addressing people’s lifestyle (Livingston et al. 2020). Of this 40%, lifetime physical activity (PA) has a crucial role in protecting against neurodegeneration and related cognitive decay (Livingston et al. 2020). It is documented that individuals with a greater physical fitness (PF) level can sustain more severe neuropathological burdens resulting in mitigated cognitive impairment (Livingston et al. 2020; Wallace et al. 2019; Erikson et al. 2018). For these reasons PA has been associated with the construct of reserve, which is defined as the individual’s ability to maintain better-than-expected cognitive and brain functions given brain insult or psychological and neurological illnesses (Stern 2000; Stern et al. 2020; Cabeza et al. 2018). Hence, it is understood that PA contributes to maintaining healthy cognition and reducing neurodegeneration (Song et al. 2022; Livingston et al. 2020; Wallace et al. 2019; Erikson et al 2018).

In the current study we investigated whether the association between physical fitness and resilience to dementia may be partially explained by the noradrenergic theory of cognitive reserve (Robertson 2013&^2014^), proposing that continuous activation (and the related integrity) of the Locus Coeruleus (LC) – noradrenergic system could be a key candidate affecting cognitive reserve and resilience capabilities. Specifically, it is hypothesized that the continuous upregulation of the LC-NA system over the lifespan might be one of the key neurobiological components for building cognitive reserve, given the marked noradrenergic decay observed in neurodegenerative diseases (Braak et al. 2011; Mather et al. 2016). In fact, it is documented that the LC-NA system degeneration is the primary marker of dementia and its loss of functionality and integrity has been associated with cognitive decline (Braak et al. 2011; Ehrenberg et al. 2017; Betts et al. 2017; Satoh et al. 2019; Dahl et al. 2020). In Robertson’s model, the noradrenergic up-regulation would be protective against the neurodegenerative dynamics due to the neuroprotective role of NA in brain (Heneka et al. 2002; Heneka et al. 2015). Supporting evidence has shown anti-oxidative (Troadec et al. 2005) and anti-inflammatory (Feinstain et al. 2002; Giorgi et al. 2020) properties of NA along with promotion of neurogenesis and synaptogenesis by increasing brain derived neurotrophic factor (BDNF) (Counts et al. 2010; Hassani et al. 2020; Traver et al. 2005; Mannari et al. 2008). Accordingly, in the last decades several studies have supported this model reporting that LC-NA system integrity was related to greater brain and cognitive health (Wilson et al. 2013; Elman et al. 2021; Jacobs et al. 2021; Plini et al. 2021; Dahl et al. 2022; Galgani et al. 2023), including better attentive and mnemonic functions both in healthy and clinical populations (Clewett et al. 2016; Dahl et al. 2019; Dutt et al. 2021; Plini et al. 2021; Prokopiou et al. 2022; Galgani et al. 2023).

**Figure1 -:**
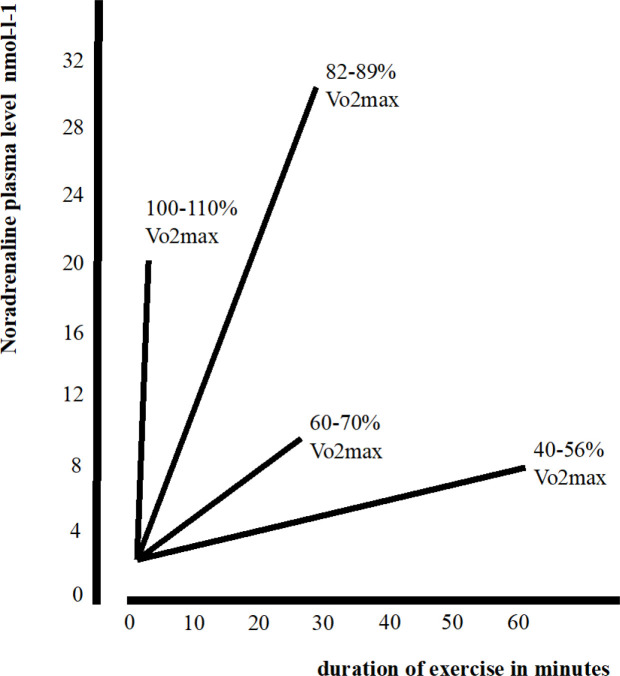
Plasma noradrenaline concentration in relation to intensity and duration of exercise. Figure adapted from Kjaer et al. 1987 with permission of Prof. Michael Kjaer

However, no studies have investigated the possible relationship between LC-NA system integrity and PA. As shown in figure 1, by increasing physical exertion, a greater amount NA is synthesized and released in response to higher oxygen demand (Kjær et al. 1987; Bekar et al. 2012; Green et al 2007; Escourrou et al. 1984; Winter et al. 2007). Maximal training intensity (between 110% and 80% of maximal oxygen consumption – Vo2max) elicits the highest NA release in comparison with lesser demanding training intensities. Indeed, the LC-NA system is involved in breath cycles and LC neurons are highly sensitive to oxygen and Co2 variations (Melnychuk et al. 2021; Melnychuk et al. 2008; Biancardi et al. 2007), and in mediating vasoconstriction and vasodilation in response to oxygen demand variations (Forense et al. 2020; Zhang et al. 2019; Bola et al. 2017; Bekar et al. 2012). Further, trained individuals show greater oxygen carrying capacity while training (Crowley et al. 2022; Milanović et al. 2015). Vo2max is a measure of oxygen uptake commonly used as a main parameter of physical fitness and cardiovascular health (Strasser and Burtscher 2018; Hanscomb et al. 2021; Xu et al. 2022; Babu et al. 2022; https://health.ucdavis.edu/sports-medicine/resources/vo2description). A large meta-analyses carried out on 102,980 individuals found that greater Vo2 max was protective against all causes of mortality, while individuals with lower physical fitness show elevated risk (Kodama et al. 2009). More recently, greater levels of Vo2max have been associated with better brain and cognitive health (Wendell et al. 2013; Hwang et al. 2017; Pantzar et al. 2018; Wallace et al. 2019; Erikson et al. 2018), greater cortical thickness (Olivo et al. 2021) and greater white matter integrity (d’Arbeloff et al. 2020; Maleki et al. 2022). Furthermore, a study conducted on 2013 healthy adults (age range 21–84) found that greater cardio respiratory fitness (CRF) was associated with greater total brain volume, and greater grey matter volume in key brain regions such as the middle temporal gyrus, the hippocampal gyrus and the orbitofrontal cortex (Wittfled at el. 2020). In the same study greater Vo2 max was also related to greater volume in the bilateral anterior cingulate cortex, an area rich in noradrenergic receptors (Palomero-Gallager et al. 2015). Correspondingly, literature on dementia reported that higher Vo2 max and overall higher levels of physical fitness were associated with better brain and cognitive health (Manning et al. 2022; Nilsson et al. 2020; Bosch et al. 2020) and stronger resilience to neurodegeneration (namely more preserved brain integrity and functioning – Olivo et al. 2021; Petkus et al. 2021; Kurl et al. 2018; Ding et al. 2018; Livingston et al. 2020).

In light of the neuroprotective effects of greater Vo2 max together with the well documented LC-NA system role in dementia progression, we investigated whether variation in Vo2max was related to the MRI signal intensity (a parameter of integrity / tissue density) of the LC-NA system. We hypothesised that greater Vo2max would be associated with greater LC signal intensity due to the neuroprotective effects of NA release. Specifically, we considered vo2max levels as a proxy measure of chronic NA up-regulation, and therefore, greater Vo2max as an outcome of the frequent and intense LC-NA system up-regulation required by strenuous PA accumulated across the lifespan. (see figure 1 adapted from Kajer et al. 1987).

To test Robertson’s model, we performed voxel-based morphometry (VBM) analyses on 41 healthy older adults provided by the Centre for BrainHealth^®^, The University of Texas at Dallas -USA (Chapman et al. 2016). First, the relationship between LC signal intensity and Vo2 max was investigated. Second, the relationship between Vo2max and other key neuromodulator seeds were investigated as a control procedure. Therefore the analyses were repeated for the Dopaminergic system using the Ventral Tegmental Area (VTA), for the Serotoninergic system using on the Dorsal and Median Raphe (DR and MR) and on for the Cholinergic system using the Nucleus Basalis of Meynert (NBM). Third, we investigated the relationship between biological brain maintenance – BrainPAD (Boyle et al. 2021) and the neuromodulatory subcortical system to explore a possible relationship between Vo2max and BrainPAD. Our final aim was to investigate the relationship between LC and a measure of high order cognitive control assessed with the test of strategic learning (TOSL) (Chapman et al. 2016).

## Methods

### Participants, Neuropsychological tests and Vo2Max assessment

The study was approved by the Institutional Review Board of the Texas Southwestern Medical Center at Dallas, University of Texas at Dallas and The Cooper Institute. All study experiments were performed in accordance with the Declaration of Helsinki and other relevant guidelines and regulations. All participants provided informed consent.

Data were provided by the Center of BrainHealth the University of Dallas Texas by Sandra Chapman and colleagues (Chapman et al. 2016). Participants were healthy older adults, 15 males and 26 females with age ranging from 60 to 72 years old. All participants underwent a preliminary screening, therefore none of them had no history of neurological or psychological illness and scored below 14 at the Beck Depression Inventory (BDI). In addition, all subjects were right-handed, not exceeded 40 of body mass index (BMI) and had as minimum education level an high school diploma. Lastly all participants’ IQ level was within the normative ranges and they scored above 26 at the Montreal Cognitive Assessment (MoCA). After the screening, participants underwent 3T high-res MRI and a neuropsychological battery which included the Test of Strategic Learning (TOSL) as primary measure for high order executive functioning. Indeed, TOSL is a tool sensitive to complex attention, memory and abstract reasoning. Subsequently, VO2max and maximum heart rate (MHR) were evaluated via electrocardiogram, a “*Lode*—*Excalibur Sport cycle ergometer, (Groningen, Netherlands) and a Jaeger Oxycon Pro, (Hoechberg, Germany)*” for Vo2max assessment. For detailed info please refer to (Chapman et al. 2016) and see table 1.

### Neuroimaging processing and analyses

41 3T T1-weighted high-res MRI images of healthy older adults (age range 60–72 - 15 males, 26 females) were processed using the standard CAT12 pipeline with a voxel size of 1mm3. The scans were modulated and oriented in MNI template and then whole brain images (MNI grey matter + white matter) were used for the formal analyses. In CAT12 a voxel-based morphometry (VBM) multiple regression model was built in order to assess the relationship between Vo2max and LC signal intensity. Age in years, gender, education, maxHR and total intracranial volume (TIV) were included as covariates and the positive relationship was tested in keeping with the main hypothesis, predicting that greater LC signal intensity would be related with higher scores Vo2 max. As a control procedure, the opposite, negative relationship was also tested. As additional control procedure, the same analyses were repeated for the neuromodulatory seed regions of the serotoninergic, dopaminergic and cholinergic systems, in order to contrast them with the noradrenergic hypothesis. The VBM analyses investigating the relationship between LC and BrainPAD and the TOSL test followed the same pipeline with the only exception being maxHR, which was not entered as a covariate.

### BrainPAD methodology

Brain Predicted Age Discrepancy (BrainPAD), calculated following Boyle et al. 2020, is an objective measure reflecting how the brain is ageing. It is similar to the previously developed indices such as Brain Gap Estimation—BrainAGE by Gaser et al. 2013, but it is computed only based on grey matter rather than white (WM) and grey matter (GM) together since GM is linearly associated with aging (Ge et al. 2002; Boyle et al. 2020). BrainPAD is obtained by calculating the discrepancy between the chronological age and the biological age of the brain defined on a healthy brain ageing trajectory of typical subjects. Boyle and colleagues defined the normal trajectory of GM ageing in healthy subjects. Then, they trained an algorithm to predict the degree of GM deterioration in relation to the chronological age in a three further populations of healthy individuals. BrainPAD provides a candidate measure of brain maintenance in terms of years of accelerated or reduced brain degeneration. Higher discrepancies between the biological brain age and chronological age are indices of abnormal ageing and poorer brain maintenance In the current study we also aimed to replicate and extend the findings reported in Plini et al. 2021, where LC signal intensity was related to better brain maintenance (lower BrainPAD scores) across 686 participants, both healthy controls, mild cognitive impairments and demented subjects.

### Neuromodulatory subcortical system ROI definition

Accurate MRI localization of the Locus Coeruleus (LC) in the human brain is still lacking of agreement (Liu et al. 2019). In the last few years, several probabilistic maps of the LC have been released (Keren et al. 2009 & 2015; Tona et al. 2017; Betts et al. 2017; Dahl et al. 2019; Liu et al. 2019; Rong Ye et al. 2020; Dahl et al. 2021; García-Gomar et al. 2022), however, these probabilistic maps, by using neuromelanin sensitive MRI sequences (neuromelanin is a pigment found in catecholaminergic neurons), described inconsistent localization and spatial extent of the LC within the MNI space. These differences reflect a relatively large anatomical variability suggesting that the LC varies across the general population both during the lifespan and in response to neurodegeneration (Yeo-Jin et al 2021; Liu et al. 2019; Keren et al. 2009). With increasing age, the LC neuromelanin signal intensity tends to shift from the rostral to the caudal portion (Liu et al. 2019; Keren et al. 2009). This process might be influenced by ageing, the degree of biological brain maintenance and even dementia progression, which is likely to exacerbate this “caudal- shifting” process. Indeed, it is acknowledged that the LC-NA system is susceptible to compensatory changes involving the caudal portion of the LC and peri- coeruleus/LC-peri-dendritic regions (Epi-coeruleus and Sub-coeruleus) extending even towards the serotoninergic nuclei (Mai and Paxinos 2012; Szot et al. 2007; Szot et al. 2006; Hoogendijk et al. 1999; Szot et al. 2000; Matchett et al. 2021; Ishimatsu et al. 1996; Janitzky et al. 2020). Therefore as outlined by Yeo-Jin Yi et al. (2021), because of these dynamics, different MRI atlases (particularly the ones developed on young populations) might not reflect the actual LC extent because of the lower strength of neuromelanin signals in young subjects. In other words, the actual LC might be underestimated because not all the LC cells (voxels) would be “*sufficiently labelled yet*” (Yeo-Jin et al 2021). Conversely, the atlases made on older populations might exclude rostral regions more likely to be labelled in younger populations. Therefore, focusing only on one of the previously published LC atlases or on a “conservative one” might be misleading in terms of generalisation of the LC spatial localization. As observed by Yeo-Jin Yi et al. (2021), the combined LC spatial localization across different subjects and conditions exceeded the typical spatial localization of an individual LC. For these reasons, in the current study, the isolation of the LC signal intensity was achieved using the “omni-comprehensive” LC mask (Plini et al. 2021) which solves the inconsistent LC spatial localization reported by previous studies (Keren et al. 2009 & 2015; Tona et al. 2017; Betts et al. 2017; Dahl et al. 2019; Liu et al. 2019; Rong Ye et al. 2020; Dahl et al. 2021; García-Gomar et al. 2022) without encroaching upon other pontine and cerebellar regions, and without crossing the walls of the 4^th^ ventricles. In figure 3c in the result section the different LC atlases can be visualized in comparison with the LC-omni-comprehensive mask. Further details can be found in supplementary materials of Plini et al. 2021 and at this link: https://www.youtube.com/watch?v=90bsA6Jqxs4). The MRI LC omni-comprehensive mask was manually developed (voxel by voxel in FSL eyes – edit mode) to carefully define a common space that included all the previous maps as to increase the likelihood of inclusion of the entirety of the LC rostro-caudal extent, and to properly account for the large individual variability documented in literature. By doing so, voxel-wise structural analyses isolating the LC-NA system were made possible in CAT12.

**Figure 2 –:**
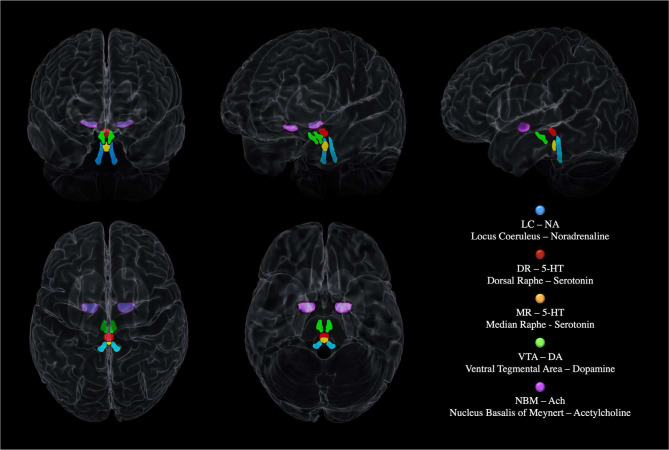
The neuromodulatory subcortical system: the main nuclei synthetizing and projecting to the cortex the main neuromodulators involved in cognition. The figure shows a 3D MRI reconstruction of the five neuromodulators’ seeds within the whole brain.

The other neuromodulatory seeds were based on previously published atlases: th MR and DR ROIs were provided by Beliveau et al. 2015, and the VTA mask was obtained by downloading the VTA MNI probabilistic map (Pauli et al. 2018) from the NeuroVault website (https://neurovault.org/ accessed on 15 December 2018). The NMB was developed on the basis of the probabilistic MNI maps of the acetylcholine cells of the Forebrain, which are provided by SPM Anatomy Toolbox 2.2c (https://www.fzjuelich.de/inm/inm1/EN/Forschung/_docs/SPMAnatomyToolbox/SPMAnatomyToolbox_node.html accessed on 15 December 2018) by Zaborszky et al. 2008 and George et al. 2011, Schulz et al. 2018, Liu et al. 2015, Kilimann et al. 2014, Koulousakis et al. 2019). These other neuromodulatory seeds were chosen on the base on their cortical and cerebellar projections. Consequently, the DR and the MR were chosen over the other Raphe nuclei because they vastly project to the cortex and the Cerebellum being the main serotoninergic nuclei of the CNS. Similarly, the VTA was chosen over the Substantia Nigra (SN) because the VTA is the main brain nucleus responsible of the cortical irroration of Dopamine (while the SN projects subcortically but not in the Cerebellum which does not shows relevant dopaminergic projections). Regarding the Cholinergic system, the NBM was chosen over the Tegmental Cholinergic Neurons, because it has the largest number of cholinergic neurons, and it projects diffusely to whole brain’s cortex (May and Paxinos 2012). Figure 2 shows the 3D MRI reconstruction of the five neuromodulators’ seeds.

### Bayesian modelling, multiple regression, ANCOVA and mediation analyses

Pearson’s and Bayesian correlation matrices were built to explore the different relationships between Vo2max, neuropsychological measures and BrainPAD with the five neuromodulators’ seeds. The average signal intensity of the clusters of voxels identified by the VBM analyses was used to better isolate each neuromodulators’ effect. The average values were extracted applying the 1mm3 binary masks of the 5 ROIs on whole brain images using FSL. In FSL the flags of “fslstats” “-k” (mask) and “-m” (output mean) were used to gather the average voxel intensities. The average signal intensity was then calculated subject-by-subject for each ROI and computed in each of the following models performed in JASP (https://jasp-stats.org/).

In the Bayesian multiple regression model Vo2max was treated as dependent variable and the five neuromodulators entered as independent covariates, in order to compare their different effects against the “*null model*”. Age, gender, education, TIV and maxHR were treated as covariates and added to the “*null model*”. The analyses followed the default JASP setting with the exception being that Bayesian information criteria (BIC) was selected in the advanced option of JASP interface.

To test whether LC signal intensity variations were congruently associated with Vo2max level, in a ANCOVA model was conducted. Vo2max was treated as a factor divided in 2 levels (Vo2max above 20 [n.22) and Vo2max below 20 [n.19]). A 20 cut-off was decided on the basis of previous literature referring to the same age range of the current sample – Kurl et al. 2018; Wendell et al. 2013; Meyers et al 2017; Kodama et al. 2009). The average LC signal intensity was entered as a dependent variable and the model was controlled for age, gender, education, TIV and maxHR. Post-hoc analyses were then Bonferroni corrected with 10000 bootstrapping.

Finally, to test potential mediation effects between Vo2max and brain maintenance, a mediation analysis with parallel multiple mediators was performed using Vo2max as a predictor (Y) and BrainPAD (X) as outcome. As multiple mediators, the average signal intensity of the five neuromodulators’ seeds were entered in parallel. The model was covaried for age, gender, education, TIV and maxHR. Standard model was set with 95% confidence intervals and 5000 bootstrap samples.

## Results

### Vo2max and Locus Coeruleus MRI signal intensity

**Figure 3 -:**
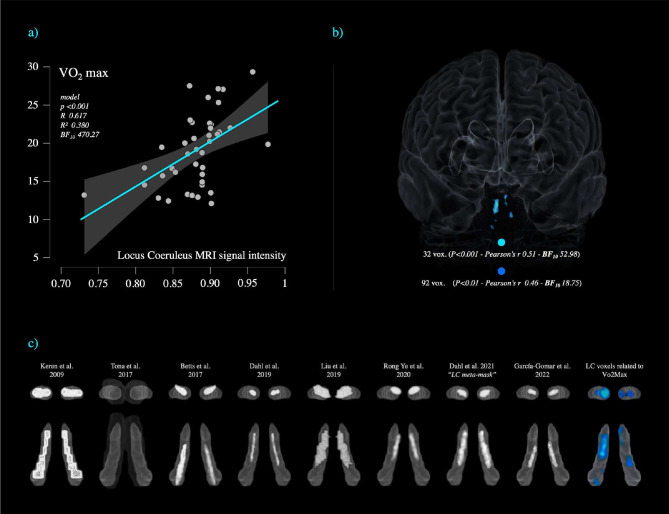
**a**) shows the relationship between vo2max and Locus Coeruleus signal intensity in a sample of 41 healthy subjects (age range 60–72). Results are covaried for Age, Gender, Education, Total Intracranial Volume and max heart rate for P<0.001*. **b**) The significant LC voxels for P<0.01 and P<0.001 clusters are displayed in a 3D-reconstruction of the brain from a midline frontal point of view. **c**) The LC clusters associated with Vo2max are shown in comparison with the previously published LC atlases and masks displayed on the LC omni-comprehensive mask by Plini et al. 2021.

As shown in table 2, the analyses confirmed a relationship between Vo2max and LC signal intensity after controlling for age, gender, TIV, education and maxHR. 92 voxels within the LC region significantly related with vo2max, i.e., greater LC signal intensity was associated with greater vo2max values. We note that a portion of the significant LC voxels in our findings overlaps the core of the previously published LC atlases and masks (Keren et al. 2009 & 2015; Tona et al. 2017; Betts et al. 2017; Dahl et al. 2019; Liu et al. 2019; Rong Ye et al. 2020; Dahl et al. 2021; García-Gomar et al. 2022), as shown in figure 3. The LC cluster survived when the statistical threshold was increased to p<0.01 and p<0.001 with a maximal Bayesian factor (BF_10_) of 55.98. Other weaker but relevant associations were observed between the DR and VTA signal intensity. A cluster of 32 voxel within the serotoninergic DR region and a cluster of 30 voxels within dopaminergic VTA region, which did not survive when the statistical threshold was raised to p<0.001. Minor associations were found for the MR and NBM nuclei. When the inverse relationships were tested, negligible results were found and none survived at p<0.01 threshold.

### Factorial analysis – ANCOVA

**Figure 4 –:**
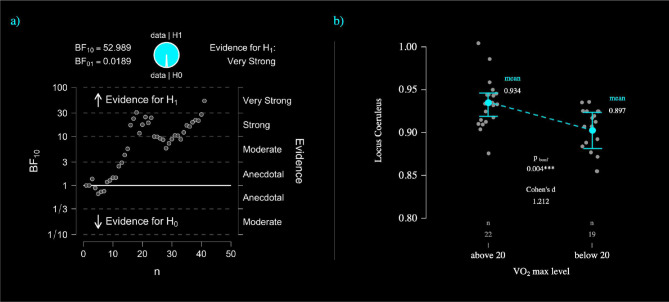
**a**) Bayesian sequential analyses for the association between Locus Coeruleus signal intensity and Vo2max. The sequential analyses reports “very strong” evidence supporting H1 (BF_10_ 52.98). **b**) ANCOVA plot displays the mean difference between the 2 levels of Vo2max (individuals above Vo2max of 20 VS individual below Vo2max of 20).

When Vo2max values were treated as a factor divided in 2 levels (Vo2max above 20 VS Vo2max below 20), the LC average signal intensity differently variated between groups. Indeed, individuals with vo2max below 20 had significantly lower LC signal intensity in comparison with individuals with vo2max above 20 (about 1.2 standard deviations - Cohens’d 1.21). This difference remained significant after Bonferroni correction (P<0.004 – refer to table 4 and figure 4b) while controlling for age, gender, education, TIV and maxHR. This analysis is consistent with the previous multiple regression model proposing the same pattern of associations.

### Brain Maintenance (BrainPAD) and Locus Coeruleus MRI signal intensity

The VBM analyses investigating the relationship between LC and biological brain maintenance revealed a similar pattern of findings. A significant relationship was found between LC signal intensity and BrainPAD; an LC cluster of 153 voxels was associated with greater biological brain maintenance. Weaker findings were observed for the NBM (52 voxels) and the DR (33 voxels). This pattern of findings replicate our previous findings relating biological brain maintenance primarily to the noradrenergic system in a larger sample of 686 subjects (Plini et al. 2021). The LC cluster survived the statistical threshold of p<0.001. All the other ROIs showed a weaker pattern not surviving the p<0.01 threshold (see table 4 and figure 4 in the supplementary materials).

### Neuropsychological Performance (TOSL) and Locus Coeruleus MRI signal intensity

In a similar vein, we observed a disproportionate link between LC and TOSL. Greater LC signal intensity related to higher scores on the TOSL, namely greater LC signal intensity was related with greater levels of high order cognitive control. A cluster of 101 voxels significantly predicted high order cognitive control performances, but did not survive multiple comparison corrections. Weaker and negligible results were observed for the other nuclei and none of them survived when the statistical threshold was increased to P<0.01 (see table 5 and figure 5 in the supplementary materials). These findings are consistent with previous work relating LC signal intensity to better cognitive performances (Clewett et al. 2016; Dahl et al. 2019; Dutt et al. 2021; Plini et al. 2021; Prokopiou et al. 2022; Galgani et al. 2023).

### Multiple regressions, mediation analyses and correlation matrices

Correlation analysis revealed no relationship between the key variables investigated. As shown in table 3, the Bayesian multiple regression model including the group of five neuromodulators, showed that the combined effect of LC and VTA signal intensity is the best model relating to vo2max, followed by LC+VTA+DR. As stand-alone variable, LC is strongest predictor of vo2max followed by the VTA. These findings corroborate the VBM analyses and are consistent with our theoretical hypothesis linking the catecholaminergic involvement in physical activity to the subcortical neuromodulator’s MRI signal intensity.

Mediation analyses showed no mediation effect for the five neuromodulators and Vo2max / BrainPAD, however, a significant direct effect between vo2max and BrainPAD was observed, leading us to infer that physical fitness might affect biological brain maintenance directly (see table 3 in supplementary materials). No other significant effects were observed for TOSL.

## Discussion

In the current study we aimed to investigate a sample of 41 healthy adults to see whether vo2max was related to LC MRI signal intensity. As a secondary aim we investigated whether LC signal intensity could be related to a measure of biological brain maintenance (BrainPAD) and to higher order cognitive functions (TOSL) as already documented in literature (Plini et al. 2021). To this end, we tested the relationship between LC-NA system MRI signal intensity and vo2max values and contrasted this hypothesis against the other main neuromodulatory systems. As we anticipated, greater vo2max values were related with greater LC signal intensity. We also observed weaker but significant relationships between Vo2max and VTA and DR signal intensity. Compared to the other neuromodulatory nuclei, greater LC signal intensity disproportionally related with higher physical fitness level, better biological brain maintenance and greater cognitive control abilities (TOSL). These findings are consistent with our proposal that vo2max might be used as a proxy index of the upregulation of the LC-NA system and (possibly) its related integrity, albeit it should be noted that other unknown confounding factors may affect the nature of this relationship. In our view, the findings support the Noradrenergic theory of Reserve suggesting that greater physical activity, as indexed by higher oxygen uptake, may contribute reserve via the integrity of the LC/NA system. However, we also demonstrated a direct effect of greater vo2max upon biological brain maintenance independent of LC involvement.

Nevertheless, these preliminary findings linking Vo2max and the LC-NA system are suggestive that greater fitness levels can affect LC integrity contributing to overall LC and brain health. Given the importance of LC integrity for predicting brain maintenance and cognition, vo2max should be further considered among the resilience factors to neurodegenerative diseases and possibly a key variable to target for curtailing or preventing LC-NA degeneration across the lifespan (Braak et al. 2011; Ehrenberg et al. 2017; Betts et al. 2017; Satoh et al. 2019; Dahl et al. 2020). Our interpretation of these findings is that greater noradrenergic tone elicited by greater physical fitness can contribute to the integrity (maintenance) of the LC-NA system via diverse mechanisms. One mechanism might involve the anti-inflammatory and anti-oxidative properties of NA, namely, increased NA release following physical exercise (Kjær et al. 1987; Winter et al. 2007) could reduce overall LC and brain inflammation (Heneka et al. 2002; Heneka et al. 2012; Traver et al. 2005; Troadec et al. 2005; Feinstain et al. 2002). Similarly, increased NA might also increase the level of BDNF while reducing biomarkers of neurodegeneration within the LC and across the brain (Omulabi et al. 2021; Counts et al. 2010; Hassani et al. 2020; Traver et al. 2005; Mannari et al. 2008).

These neurobiological mechanisms might also explain the short- and long-term repercussion of physical activity on cognitive performances (Basso and Suzuki 2017; van Dogen et al. 2016; Blomstrand & Engvall 2020; Bosch et al. 2020; Nilsson et al. 2020; Winter et al. 2007), as several studies described enhanced cognitive functions immediately after physical exercise and better cognitive performances in well trained individuals (Wendell et al. 2013; Hwang et al. 2017; Pantzar et al. 2018; Wallace et al. 2019; Erikson et al. 2018). In a work from Nilsson and colleagues (Nilsson et al. 2020), the increased BDNF plasma levels following acute exercise were associated with greater cognitive training gains over a period of 12 weeks. Furthermore, another study by Engerof and colleagues (Engerof et al. 2018) found the regular physical activity led to greater bioavailability of BDNF resulting in greater brain volumes. These neurobiological processes are mediated by the LC-NA system which both underpin adaptation to physical stress and cognitive functioning (Kjær et al. 1987; Winter et al. 2007; Bekar et al. 2012; Green et al 2007; Jimenez et al. 2007; Escourrou et al. 1984; Robertson 2013&^2014^, Mather 2016). This interconnection was well presented empirically in a multimodal MRI study by Mather and colleagues (Mather et al. 2020), where several sets of isometric handgrip contractions for 18 seconds before cognitive tests were related to greater LC phasic activity during an fMRI attentional task, resulting in faster reaction times compared to control condition. In addition, Mather and colleagues, also reported that greater structural LC MRI contrast was related to better attentional control. Moreover, a previous study by Segal and colleagues (Segal et al. 2012) showed that 6 minutes of physical activity at 70% of vo2max enhanced memory consolidation of stimuli presented earlier both in MCI and healthy individuals. In parallel with enhanced memory, the groups which trained after stimuli presentation showed also significantly higher NA levels relative to controls. This interdependence between physical activity, cognition, catecholamines and BDNF levels was earlier demonstrated by Winter and colleagues (Winter et al. 2007) on a study on 27 healthy young subjects. Winter and colleagues measured catecholamines and BDNF plasma levels at baseline, after physical exercise and after a subsequent learning task. They found that, compared to rest- and moderate physical activity-conditions, only intense physical activity (6 min. running) elicited greater catecholamine and BDNF release and that these levels predicted greater immediate learning, and greater retention both at intermediate (1week) and long term (8–10months) stages. These studies, corroborate preceding evidence revealing 5-fold NA increase for 2 hours following exercise (Chatterton et al. 1996; Brenner et al. 1997; Brenner et al. 1998; Urhausen et al. 1995; Kjær et al. 1987), and well support the concept that enhanced NA release due physical activity might subsequently influence cognitive performance via LC-NA activity. In addition, from the reserve/resilience point of view, the long-term beneficial effect of PA on LC-NA system might also involve the chronic over-expression of neuropeptide Galanin (O’Neal et al. 2001; Van Hoomissen et al. 2004; Holmes et al. 2006). Indeed, it has been reported a dose-dependent PA-Galanin expression within the noradrenergic neurons (O’Neal et al. 2001; Holmes et al. 2006; Murray et al. 2010), which ameliorated stress-induced anxiety-like behaviour in murine models (Tillage et al. 2020). Other studies also reported a protective role PA-Galanin over-expression in maintenance of dendritic density and neurite outgrowth (Nowacka-Chmielewska et al. 2022; Sciolino et al. 2015; Holmes 2014; Hobson et al. 2013). Similarly, this effect might be combined with PA-BDNF levels, which were associated with increased LC dendrite density (Nakai et al. 2006; Matsunaga et al, 2004) as well as promoting neurogenesis in key brain structures such as the Hippocampus (Liu and Nusslock 2018).

**Figure 5 -:**
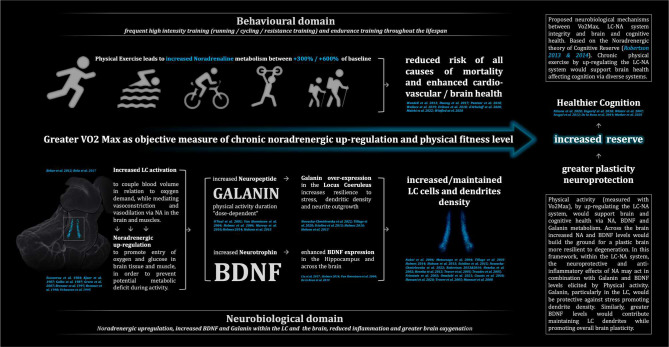
proposed neurobiological mechanisms between Vo2Max, LC-NA system and brain and cognitive health. Based on the Noradrenergic theory of Cognitive Reserve (Robertson 2013 & 2014). Physical activity (measured with Vo2Max), by up-regulating the LC-NA system, would support brain and cognitive health via NA, BDNF and Galanin metabolism. In this framework, the neuroprotective and anti-inflammatory effects of NA may act in combination with Galanin and BDNF levels elicited by Physical activity. Galanin, particularly in the LC, would be protective against stress promoting dendrite density. Similarly, greater BDNF levels would contribute maintaining LC dendrites while promoting overall brain plasticity.

The above extant literature might suggest that vo2max variations can affect LC-NA integrity (maintenance) in humans, possibly influencing the degree of LC-NA resilience to neurodegeneration via several pathways including NA and following BDNF and Galanin metabolism (Nakai et al. 2006; Matsunaga et al, 2004; Holmes 2014; Nowacka-Chmielewska et al. 2022; Gibbons et al. 2023 – **in** figure 5 **we summarise the aforementioned mechanism**). Our preliminary findings show that vo2max varies with signal intensity, disproportionately within the structure of the LC, and highlights a potential mechanism in which physical fitness builds Reserve through the neuroprotective role of NA metabolism across the central nervous system (Robertson, 2013). However, it is worth mentioning that, as emerged both from VBM analyses and Bayesian modelling, our findings outline how other main neuromodulators can have a relevant role in relationship to vo2max and PA. Indeed, the observed associations between the dopaminergic VTA and serotoninergic DR and MR with vo2max are suggestive that greater physical fitness might be related with greater structural integrity of such nuclei. Indeed, in a similar way to NA but in minor extent, evidence shown increased 5-HT and DA following physical training (Heijnen et al. 2016; Cordeiro et al. 2017; Kjær et al. 1987; Bekar et al. 2012; Green et al 2007; Escourrou et al. 1984; Winter et al. 2007). Consistently, animal studies also shown greater concentrations both of 5-HT and DA within the cortex and the brainstem immediately after exercise (Heijnen et al. 2016; Dey et al. 1992; Meeusen and De Meirleir 1995; Foley and Fleshner 2008) implying greater integrity and functionality of such neuromodulatory systems might result in chronic (Foley and Fleshner 2008; Heijnen et al. 2016; Meeusen 2005; Dey et al. 1992). These findings together with our results are indicative that Vo2max levels might affect 5-HT and DA nuclei integrity in humans for the same mechanisms discussed above. Nevertheless, the overall monoaminergic involvement in response to PA might be considered as factor capable to shape brain and cognitive health, and that greater Vo2max level can simultaneously stimulate Brainstem nuclei, possibly resulting in greater integrity via the neuroprotective role of NA as described by Robertson (2013, 2014). It is possible that the neuroprotective role of NA on dopaminergic and cholinergic neurons as described in literature may also partially explain the nature of the association between Vo2max and other neuromodulatory nuclei (Zhu et al. 2018; Heneka et al. 2002; Heneka et al. 2015; Troadec et al. 2005; Feinstain et al. 2002; Giorgi et al. 2020; Counts et al. 2010; Hassani et al. 2020; Traver et al. 2005; Mannari et al. 2008). However, it worth mentioning the described noradrenergically mediated mechanisms might interact with other neurochemical dynamics, or even that the observed associations may be entirely dependent on different neurobiological domains related to physical activity, such as lactate metabolism for instance (Hashimoto et al. 2021; Cai et al. 2022; Xue et al. 2022).

### Limitations

The main limitation of the current preliminary study is the small sample size; a larger sample and a longitudinal design would provide more accurate casual relationships among these variables and would enable an understanding of possible trajectories across time. The number of participants may also explain why our significant associations did not survive multiple comparison corrections and the lack of mediation effects. However, our sample size is similar to other studies investigating vo2max in relationship to brain and cognitive variables (Manning et al. 2022; Nilsson et al. 2020; Bosch et al. 2020), and also matched the size of other relevant studies whose used the same methodology than ours (Olivo et al. 2021; Petkus et al. 2021). Furthermore, we have also enhanced the validity of our design by performing thorough control analyses, contrasting 9 different hypotheses to the main hypothesis, and systematically covarying for relevant confounding factors such as age, gender, education, total intracranial volume, and max heart rate. These control analyses and the employment of both null hypothesis statistical testing and Bayesian modelling, strengthens the reliability of our observations.

Another constraint involves the methodological limitations of VBM analyses in retrospective studies which should be taken into account while considering these findings. Post-mortem histological investigations would offer more precise quantifications about the relationship between vo2max and the integrity (maintenance) of the neuromodulatory subcortical system. However, within these limitations, our findings are consistent with previous *in-vivo* evidence using similar methodology (Plini et al. 2021; Dutt et al. 2021; Olivo 2021; Petkus et al. 2021).

Lastly, it should be noted that the current study did not control for other potential confounds, such as genetic variables, ethnicity, body parameters (ratio between body fat and lean mass) and dietary behavior which can affect Vo2max level (Santisteban et al. 2022; Williams et al. 2017; Nightingale et al. 2016; Bacon et al. 2013; Muoio et al. 1994), LC variation and brain and cognitive health as well. It is important to note that this is a preliminary investigation based on a relatively small sample focused on VBM analysis of circumscribed areas of the brainstem. Although, many of these findings are consistent with the literature, further (longitudinal) investigations in larger samples are required. Nevertheless, the current findings show consistency between multiple regression and Bayesian approaches.

## Conclusions and clinical implications

This is the first preliminary evidence linking *in vivo* LC-NA system integrity to Vo2max in healthy older subjects. These findings provide a possible explanation behind the neurobiological mechanisms connecting physical fitness to improved cognitive functions and resilience to neurodegenerative diseases. These novel and unique results shed light on possible neurobiological dynamics underpinning the relationship between physical fitness and brain health, whilst being consistent with Robertson’s theoretical framework positing the noradrenergic system as a key neuromodulatory basis of Reserve. However, it should be noted that the integrity of the LC-NA system may be the factor which leads to a higher exercise capacity (greater vo2max), and that the current study was not designed to address this. Therefore, future studies, using physical exercise to specifically target the LC-NA system, should aim to replicate the current findings, focusing particularly on prospective investigations including biomarkers, neuropsychological testing, body metrics and other life-style factors (including dietary behaviour). The current study provides additional evidence on the growing literature outlining the pivotal role of physical activity in matter of brain maintenance and cognitive health particularly in the face of neurodegenerative diseases (Matta Mello Portugal et al. 2013; Tyndall et al. 2018; Barnes et al. 2018; Livingston et al. 2020; Basso et al. 2022). This study adds support for preventative strategies focused on physical training protocols as a practical tool for increasing brain and cognitive health and protecting against neurodegeneration (Livingston et al. 2020; Batouli & Saba 2017; Freberg & Taglialatela 2022; Gibbons et al. 2023). Indeed, several studies reported that vo2max is effectively trainable using various methodologies at different ages in healthy populations (Gormley et al. 2008; Ozaki et al. 2013; Murawska-Cialowicz et al. 2015; Scribbans et al. 2016; Menz et al. 2019; Wen et al. 2019). Physical activity interventions also can significantly improve cognitive and brain health (Chapman et al. 2016; Kovacevic et al. 2020; Nilsson et al. 2020; Blomstrand & Engvall 2020; Zhu et al. 2021; Liu et al. 2021; Upadhyay et al. 2022; Basso et al. 2022), while evidence showed that in even MCI and early stages of dementia physical exercise can alleviate cognitive impairment, possibly slowing down disease progression (Anderson et al. 2011; Segal et al. 2012; Arcoverde et al. 2014; Reiter et al. 2015; Varma et al. 2017; Barnes & Corkery 2018; Yu et al. 2020).

## Figures and Tables

**Table 1:** shows the key sociodemographic, neural indices and neuropsychological characteristics of the sample tested. Key: TIV, total intracranial volume; MaxVo2 (maximal oxygen consumption); MaxHR (maximum heart rate); BrainPAD (Brain predicted age discrepancy (biological maintenance index); TOSL (test of strategic learning).

	age		education		TIV		MaxVO2		MaxHR		BrainPAD		TOSL	
	female	male	female	male	female	male	female	male	female	male	female	male	female	male
n.	26	15	26	15	26	15	26	15	26	15	26	15	26	15
Mean	63.846	64.000	16.692	16.667	1356.84	1561.87	18.478	23.491	158.000	146.133	−1.800	−2.090	5.346	4.733
SD	3.552	3.140	2.035	1.447	91.432	110.200	2.972	4.380	13.345	14.352	7.878	11.245	1.832	2.374
Min.	57.000	58.000	12.000	14.000	1195.16	1401.53	14.350	14.050	125.000	125.000	−13.048	−30.205	2.000	1.000
Max.	71.000	70.000	18.000	18.000	1535.43	1792.00	23.000	29.630	181.000	169.000	12.398	16.243	10.000	10.000

**Table 2:** shows the results for the VBM multivariate linear regression analyses testing the positive relationship between the five ROIs and vo2max.

Neuromodulatory System	side	MNI coordinates			peak T value^a^	peak Z score^b^	peak cluster Ke^c^	p value uncorr^d^	FWE^e^	FDR^f^	total number of voxels for p<0.05 with max BF_10_^g^
		x	y	Z							
**vo2 max**											
Locus Coeruleus**	right	4	−38	−22	3.72	3.39	65	0.000	1.000	1.005	92 (BF_10_ 52.98)
Dorsal Raphe*	/	0	−28	−10	2.77	2.61	5	0.004	1.000	1.005	32 (BF_10_ 9.173)
Median Raphe	right	2	−34	−20	2.01	1.95	6	0.632	1.000	1.005	6 (BF_10_ 2.233)
Ventral Tegmental Area*	right	−2	−24	−18	2.61	2.47	3	0.446	1.000	1.005	30 (BF_10_ 80.35)
Nucleus Basalis of Meynert	left	−16	−8	−8	1.91	1.86	3	0.870	1.000	1.005	3 (BF_10_ 0.150)

The results across the n.41 healthy subjects are covaried for age, gender, total intracranial volume, education and maximal heart rate (maxHR). The table reports the significant clusters of voxels predicting vo2max values for the statistical threshold of p<0.05. Bayesian Factors (BF_10_) are reported as parameter of strength in brackets. Cluster of voxels surviving p<0.01 are marked with *. Cluster of voxels surviving p<0.001 are marked with **. No clusters survived multiple comparison corrections (FWE).

a) Peak T value: T value of the most significant cluster of contiguous voxels

b) Peak Z-score: Z-score of the most significant cluster of contiguous voxels

c) Peak cluster Ke: number of voxels of the most significant cluster of contiguous voxels

d) P value uncorrected

e) FWE = family wise error correction value

f) FDR = false discovery rate correction value (q)

g) Total number of voxels outcoming in the ROI including all clusters of contiguous voxels (in brackets are reported Bayes Factors)

**Table 3:** reporting the results of the Bayesian multiple regression model investigating the associations between the five ROIs of the neuromodulatory subcortical system and vo2max by covaring for age, gender, education, total intracranial volume and max hearth rate.

Bayesian Multiple Regression Model Comparison – vo2max and the neuromodulatory subcortical system					

Models	P(M)	P(M|data)	BF_M_	BF_10_	R^2^

Null model (incl. age, education, gender, TIV, MaxHR)	0.167	8.737e-4	0.004	1.000	0.414
LC + VTA	0.017	0.162	11.381	1850.837	0.661
LC + DR + VTA	0.017	0.109	7.232	1249.826	0.685
DR + VTA	0.017	0.101	6.617	1154.276	0.653
LC	0.033	0.082	2.596	470.274	0.604
VTA	0.033	0.063	1.964	362.961	0.599
LC + VTA + NBM	0.017	0.056	3.477	637.007	0.674

*Note.* All models indude age, education, gender, TIV, MaxHR. Table displays only a subset of models; to see all models, select “No” under “Limit No. Models Shown”.

**Table 4:** reporting the results of the ANCOOVA model treating vo2max as factor divided in 2 levels (above 20, below 20) and Locus Coeruleus average signal intensity as a dependent variable while controlling for age, gender, education, TIV and maxHR. Locus Coeruleus signal intensity significantly differs (Cohens’s d – 1.21) between the 2 groups (P<0.01 Bonferroni corrected).

ANCOVA – Locus Coeruleus signal intensity and VO2Max level					

Cases	Sum of Squares	df	Mean Square	F	p

VO2Max Level	0.011	1	0.011	9.847	0.004
gender	0.001	1	0.001	1.123	0.297
age	6.902e-4	1	6.902e-4	0.599	0.444
education	4.150e-6	1	4.150e-6	0.004	0.953
TIV	0.001	1	0.001	1.067	0.309
MaxHR	4.896e-4	1	4.896e-4	0.425	0.519
Residuals	0.039	34	0.001		

Bootstrapped Post Hoc Comparisons - Vo2Max level								

	99% bca† CI							
			
	Mean Difference	Lower	Upper	SE	bias	t	Cohen’s d	P_bonf_

Above 20	0.039	0.014	0.079	0.012	−2.687e-4	3.138	1.212	0.004***

*Note.* Type III Sum or Squares

** p < .01

*Note.* The model is controlled for the effect of age, gender, education, TIV and maxHR.

*Note.* Mean Difference estimate is based on the median of the bootstrap distribution.

*Note.* Cohen’s d does not correct for multiple comparisons.

*Note.* Bootstrapping based on 1000 successful replicates.

*Note.* Results are averaged over the levels of: gender

† Bias corrected accelerated

## Data Availability

The study was approved by the ethical review board of the University of Texas Southwestern Medical Center at Dallas, University of Texas at Dallas and The Cooper Institute. All participants were informed about the study protocol and provided informed written consent prior to scanning.” The datasets used and/or analysed during the current study available from author Jeffrey S. Spence, Center for BrainHealth, University of Texas at Dallas, on reasonable request. The Locus Coeruleus omni-comprehensive mask and the nifty images for significant clusters are available on request. Contact Dr. Emanuele RG Plini (plinie@tcd.ie / emanuele.rg.plini@gmail.com)
